# Identification of a Conserved Temporal Molecular Response Signature to CDK4/6 Inhibition Through Multi-Omics Integration Across Cancer Cell Lines

**DOI:** 10.3390/ijms27198507

**Published:** 2026-09-24

**Authors:** Anna C. Hogger, Ting Hong, Klaus Mantwill, Nadine Gaisa, Jürgen E. Gschwend, Roman Nawroth

**Affiliations:** 1 Department of Urology, TUM Klinikum Rechts der Isar, Technical University of Munich, Ismaninger Straße 22, 81675 München, Germany; 2 Institute of Pathology, University Hospital RWTH Aachen, RWTH Aachen University, Pauwelsstraße 30, 52074 Aachen, Germany; 3 Institute of Pathology, University Hospital Ulm, University of Ulm, Albert-Einstein-Allee 23, 89081 Ulm, Germany

**Keywords:** CDK4/6 inhibitor, palbociclib, molecular marker, response mechanism, G0/1 arrest, M-phase

## Abstract

Treatment of metastatic breast cancer with CDK4/6 inhibitors in combination with hormone ablation is the current first-line standard of care for this indication, and the therapeutic activity of these inhibitors has been demonstrated in various tumor entities. However, the efficacy of CDK4/6 inhibitor monotherapy is limited and requires combination with suitable additional inhibitors. Delineating the molecular pathways underlying treatment response may provide a rationale for personalized combination therapies that target deregulated pathways upstream of the core signature of response. We applied a comprehensive cross-omics approach to identify a conserved molecular response to CDK4/6 inhibition. Integration of transcriptomic, proteomic, and functional genomic data across multiple cancer models enabled us to refine a previously proposed multi-step model of response to the CDK4/6 inhibitor palbociclib. Initiation of cell-cycle arrest and inhibition of DNA replication is followed by activation of signaling events that suppress mitotic transcriptional programs. The identified panel of highly conserved candidate biomarkers warrants future experimental and clinical validation.

## 1. Introduction

Deregulation of the Cyclin D–CDK4/6–RB pathway represents one of the most common oncogenic events across human malignancies. Inhibition of cyclin-dependent kinases 4 and 6 (CDK4/6) in combination with endocrine therapy is the standard of care for the treatment of hormone receptor–positive, HER2-negative, advanced, or metastatic breast cancer [1]. Despite this clinical success, therapeutic efficacy remains heterogeneous in many other tumor entities, and both intrinsic and acquired resistance continue to limit durable clinical responses [2,3,4]. To date, three CDK4/6 inhibitors—palbociclib, abemaciclib, and ribociclib—have been approved by regulatory agencies. However, these compounds exhibit limited monotherapeutic efficacy, which can be overcome by combination with other therapies [5,6,7].

In the present study, we focused on palbociclib (PD), which, in combination with endocrine therapy (ET), was the first in-class approved CDK4/6 inhibitor approved by both the FDA and EMA [1,8,9]. The objective response rate to PD + ET ranges from 25 to 55.3% [10,11,12]. Real-world data have shown that PD in combination with either aromatase inhibitors or letrozole significantly improves both overall survival and progression-free survival of patients [13,14].

One strategy to improve patient stratification is to investigate molecular processes that are consistently activated or suppressed in response to CDK4/6 inhibition across different cellular contexts. These fundamental mechanisms might help to improve the efficacy of CDK4/6 inhibitors whilst identifying promising candidate biomarkers for future clinical applications. Previously, we have shown that the transcriptional response to palbociclib in the bladder cancer cell line T24 is regulated by temporally coordinated molecular changes in a multi-step mechanism [15]. The present study builds on this model and aims to investigate whether this temporal organization is conserved across different tumors.

Dysregulation of cell-cycle regulators such as cyclin-dependent kinases (CDKs) and cyclins is a common driver of aberrant cell proliferation across various cancers [8]. Beyond cell-cycle progression, CDK4/6 modulates cellular metabolism and lysosomal biogenesis, promotes an anti-tumor immune response, and induces senescence. Accordingly, several hundred molecular targets have been validated for CDK4/6 activity [16]. One of these targets is the retinoblastoma 1 (RB1) protein, which, in its dephosphorylated form, binds members of the E2F transcription factor family and represses cell proliferation [8,15]. Upon phosphorylation by CDK4/6, RB1 releases E2F, activating the expression of genes required for S-phase transition [17].

Although RB1 is required for the response to CDK4/6 inhibition, the temporal molecular events that characterize this response across different cancer types remain poorly defined. Therefore, we performed a comprehensive multi-omics gene-list comparison of published transcriptomic, proteomic, and functional genomic datasets to identify conserved temporal response programs and robust candidate biomarkers.

We systematically searched for molecular alterations in response to PD that are consistently observed across multiple cancer cell lines, experimental platforms, and treatment durations. First, transcriptomic datasets were curated and integrated to identify conserved temporal response patterns. These were subsequently cross-referenced against independent, curated proteomic datasets as well as previously published loss-of-function and gain-of-function CRISPR screens.

Based on a transcriptomics time-course analysis over 48 h, we have previously shown that the response to the CDK4/6 inhibitor palbociclib is organized in a multi-step process and identified temporally coordinated molecular responses to therapy [15]. By integrating complementary omics levels, we extend and build upon these data by using them as a framework to interpret a broader collection of datasets and provide a mechanistic context that represents conserved components of the effect of CDK4/6 inhibitors across various cancer cell lines and experimental platforms. This analysis revealed that the initial response to PD treatment is primarily induced by initiation of cell-cycle arrest and inhibition of DNA replication. Analyzing the temporal kinetics uncovered signaling pathways involved in the inhibition of mitotic gene expression programs as a secondary step. In summary, the results of this study support and refine the previously published multi-step model of response to PD and might also provide evidence for identifying biomarkers that allow for monitoring therapy response.

## 2. Results

### 2.1. A Transcriptomics Meta-Analysis Identifies Conserved DEGs Across Different Tumor Cell Lines and Treatment Duration

The aim of this analysis was to identify highly conserved differentially expressed genes (DEGs) in response to the CDK4/6 inhibitor palbociclib. We hypothesized that identification of recurring DEGs by integration of published transcriptomic data would result in a robust selection of DEGs and pathways that are central to the response to PD treatment. We first performed a comprehensive meta-analysis of published transcriptomic data. Inclusion criteria were monotherapy with PD for 6–72 h and available, preprocessed differential expression data (log2FC, FDR). In total, we identified seven independent studies beyond a dataset from our group that provided data from 17 different cancer-derived cell lines and five time points after treatment, resulting in 27 single datasets (Figure 1A) [15,18,19,20,21,22,23,24].

The combined datasets yielded 29,384 unique DEGs (Figure 1B). To ensure data quality, DEGs were filtered for an absolute log2 fold change in expression compared to the untreated 0 h control of >1 and a false discovery rate (FDR) of <0.05. This resulted in a refined pool of 4520 DEGs and, after removing redundant gene isoforms, a final set of 4399 unique DEGs.

### 2.2. The Transcriptional Response to PD Is Organized into Three Consecutive Phases

To provide a comparative overview, we generated a heatmap (Figure 2A) illustrating the proportional overlap of DEGs across all published datasets. The overlap was calculated relative to the smaller dataset in each pairwise comparison. Datasets were organized primarily by duration of PD treatment and secondarily by descending mean percent overlap in DEGs. Jaccard similarity for each pairwise comparison is additionally provided in Supplementary Table S1 and Figure S1.

The mean percent overlap of each dataset with all other datasets (top annotation boxplots) revealed a broad similarity. While most datasets display an intermediate median overlap with all other datasets (around 50%), the high variance in these boxplots reflects structured transcriptomic divergence based on cell line and duration of treatment. Another reason for the observed high variance in overlaps is a subset of data (T24_8h, HCC1806_24h, 5637_24h, J82_24h, and AsPC-1_72h) that exhibits exceptionally low overlap with the broader collection of datasets. The lack of overlap correlated with the low number of DEG counts in these datasets. Nevertheless, a small number of DEGs alone (top annotation bar plot) did not necessarily result in low levels of overlap, since these datasets still shared a significant proportion of conserved genes with other datasets; thus, even small sets of DEGs contain biologically conserved and functionally relevant transcriptional changes.

In general, we observed high degrees of overlap among datasets with similar durations of PD treatment, namely within the 6, 24, and 72 h samples. Additionally, there are overlaps between the 6 h and certain 24 h samples, as well as between 24 and 72 h samples.

Notably, these overlaps across different time points were observed primarily when datasets originated from the same publication. Consequently, we performed a PERMANOVA analysis based on Jaccard distances to assess the influence of the individual publication on dataset DEG composition. Because cell lines were nested within publications, we modeled this structure using a nested PERMANOVA (Publication/Cell line). The origin of the dataset was a significant predictor of DEG composition (R^2^ = 0.38, *p* = 0.004). This analysis suggests publication-specific differences, likely caused by methodological differences across publications. A principal coordinates analysis (PCoA) revealed that this effect mostly focuses on the datasets from Hafner et al. (2019) and displayed distinct clustering of these 6 and 24 h datasets (Figure 2B). No clustering by publication or duration of PD treatment was apparent among the remaining datasets.

Cell lines tested for their effect within publications did not explain additional variance (R^2^ = 0.26, *p* = 0.99), indicating that DEG compositions showed some consistency across cell lines when derived from the same publication. However, this analysis is based on Jaccard distances over the full DEG list of each sample and is, therefore, most sensitive to broad compositional shifts. It does not rule out cell line–specific effects in particular genes or pathways.

While the initial analysis revealed heterogeneity in dataset size (Figure 2A, top annotation bar plot), stratification by treatment duration uncovered consistent patterns in the number of DEGs (Figure 2C). As also indicated in the heatmap (Figure 2A), the datasets clustered into three distinct temporal subgroups: T-early (6–8 h), with an average of 83 DEGs (SD: 51); T-intermediate (24 h), with an average of 353 DEGs (SD: 403); and T-late (48–72 h), with an average of 522 DEGs (SD: 536). This finding supports our previously proposed and published model regarding the therapy response to CDK4/6, which is organized into three distinct and temporally coordinated phases: first, the initiation phase of response (6–8 h); second, a phase of plasticity (24 h), where cellular commitment to either sustained response or acquired resistance occurs; and third, the phase spanning 48–72 h, likely reflecting the status in which a molecular response has been manifested [15].

To identify a consensus signature for each phase, DEG lists of all datasets within temporal subgroups were combined (Figure 2D, Supplementary Table S2). While the fold change in the average number of DEGs from T-early to T-mediate is 3- to 4-fold, it increased for the combined DEG lists to 7-fold. This is because the total number of unique DEGs in the combined sets (Figure 2D) significantly exceeded the DEG counts in the individual datasets (Figure 2C). This difference indicates that while different cell lines exhibit similar levels of transcriptional change (Figure 2C), the specific composition of affected genes is largely cell line–specific.

Next, we determined the number of intersecting DEGs between T-early, T-intermediate, and T-late (Figure 2D, Supplementary Table S2). This analysis revealed a core consensus signature of 138 DEGs that remained differentially expressed across all time points (a specific DEG is present in at least one dataset of T-early, T-intermediate, and T-late). The 138-gene intersection showed a 29-fold change enrichment over the expected overlap by chance (multi-set intersection testing: FDR = 2.4 × 10^−163^ (Supplementary Table S14)).

To account for possible overlaps caused by shared experimental procedures or processing pipelines, we excluded nine candidates that showed consistency only across time points and datasets derived from the same publication (Supplementary Table S3). The remaining 129 DEGs are differentially expressed across different cell lines during the early, intermediate, and late phases of therapeutic response to PD (T-consensus).

Considering the partially high overlap rates between individual datasets in Figure 2A, the number of common DEGs for all three subgroups was comparatively small (Figure 2D). Combined with the indicated cell line–specific transcriptional responses, this finding suggests that many overlapping DEGs are specific to certain cell lines and, therefore, are unlikely to represent general mechanisms underlying sensitivity or resistance to PD.

To further assess the robustness of individual DEGs, we calculated their recurrence across all datasets. The frequency distribution shows that the majority of all DEGs (70%) were identified in only a single dataset unrelated to response or resistance to therapy (Figure 2E, Supplementary Table S4), indicating that most transcriptional changes are context-specific rather than broadly conserved. Notably, no DEG was differentially expressed in all 27 datasets.

In summary, while there are differences in the number of DEGs, experimental conditions, and, most importantly, cell line–specific transcriptional regulation after PD treatment, we identified a subset of 129 genes that are differentially expressed independently of cell line or duration of PD treatment.

### 2.3. DEGs from T-Consensus Are Functionally Connected and Involved in S-Phase Progression, DNA Replication, and Repair

The 138 DEGs that showed consistent differential expression across T-early, T-intermediate, and T-late (Figure 2D) were utilized as input for a STRING protein–protein interaction (PPI) network analysis. Nine DEGs were excluded because they were only represented across datasets derived from the same publication. Six more DEGs had to be excluded due to the absence of STRING identifiers (Figure 3A). They comprised a collection of poorly described genes and long non-coding RNAs. In total, 89 of the remaining 123 genes formed a cohesive functional network, while 44 remained as singletons. We manually added CDK4, CDK6, and RB1, as they are the primary and well-established targets and core mediators of CDK4/6 inhibition. Their inclusion provides a fixed anchor point, allowing the network to be interpreted in the context of established mechanisms of response to CDK4/6 inhibition. Furthermore, we performed targeted expansion using the “expand network” function of the Cytoscape stringApp (v2.2.0) to identify proteins that link singletons to the existing network. The refined network consisted of 100 interconnected nodes and 33 singletons (Figure 3B). After functional annotation and clustering of nodes, four major clusters were identified, including proteins involved in DNA replication, DNA repair, cytokinesis, and cell-cycle control (Figure 3B). These findings were supported by a pathway enrichment analysis for the original 129 candidates of T-consensus, which demonstrated that significantly enriched pathways across all time points (6–72 h) were predominantly centered on cell-cycle progression, DNA replication, and DNA repair machinery (Figure 3C).

### 2.4. CDK4/6 Inhibition Induces a Thorough Cell-Cycle Arrest by Impairment of G1/S-Transition and Suppression of the Mitotic Gene Expression Program

To gain a better understanding of the processes within the three phases of response to PD, an intersection analysis was performed for T-early, T-intermediate, and T-late. T-early comprised seven datasets from two publications that showed a consensus signature of eight downregulated DEGs (T-early consensus: CDT1, DTL, E2F1, MCM2, ORC1, PCNA, WDR76, and ZNF367) (Figure 4A, Supplementary Table S5). An additional five downregulated genes were found to be differentially expressed exclusively within the datasets published by Hafner et al. (2019) (ATAD2, CDC6, HELLS, MCM10, and RBL1) [18]. We visualized the eight DEGs present in all T-early datasets alongside the additional five genes. Although these five genes were not detected in all early studies, they share close functional relationships with the eight core candidates. Functionally, the encoded proteins are primarily involved in the initiation of DNA replication (Figure 4B). This observation was further supported by a pathway enrichment analysis, which showed significant overrepresentation of G1/S-phase transition and DNA replication and repair, as expected (Figure 4C).

While data from Hafner et al. (2019) and Hong et al. (2024) share common DEGs and have a broad alignment of functionally affected pathways, the higher DEG count (Figure 2C) in the dataset from our group suggests further underlying information [15]. To ensure that focusing exclusively on overlapping DEGs does not overlook relevant biological insights, we performed a separate in-depth analysis of our 8 h early-response dataset.

Visualization of the top DEGs as a protein–protein interaction network (Supplementary Figure S2) confirmed the suppression of canonical cell-cycle pathways and upregulation of the immune response. However, this analysis further identified several functional clusters that were not previously characterized in the context of CDK4/6 inhibition. We identified the Ephrin receptor family as a potential therapeutic target and validated this result by targeting Ephrin receptors and CDK4/6 using small-molecule inhibitors against both in parallel (Supplementary Figure S3). Analysis of cell death showed synergistic inhibition of cell growth, using the ZIP-synergy score, suggesting Ephrin receptors to be a novel target for a combination therapy with PD.

To maintain analytical stringency within T-intermediate, the cell lines HCC1806, 5637, and J82 were excluded due to limited DEG counts and lack of intersection with the other datasets within the group (Figure 2A,C). Among the remaining 12 datasets, a consensus signature of 22 consistently downregulated DEGs (T-intermediate consensus) was identified (Figure 4D, Supplementary Table S6). The STRING PPI network analysis revealed that 14 of these downregulated 22 DEGs formed a highly interconnected network comprising the regulation of cytokinesis, spindle assembly, and spindle–kinetochore interaction (Figure 4E). Consistent with these findings, pathway enrichment analysis highlighted a significant overrepresentation of processes related to the M-phase (Figure 4F).

For T-late, the cell line AsPC-1 was excluded as it had only two significant DEGs, which is insufficient for intersection analysis. Among the four remaining datasets, three genes (T-late consensus: LMNB1, MXD4, and PRIM1) were represented in all of them (Figure 4G,H, Supplementary Table S7). This analysis reveals a molecular response that follows tightly regulated time kinetics. The early response is centered around the inhibition of G1/S-transition and initiation of DNA replication. This initial response is followed by a secondary inhibition of an M-phase-related gene expression program.

### 2.5. CDK4/6 Inhibition Results in Primary Inhibition of G1-S-Phase Transition and Secondary Inhibition of the Mitotic Gene Expression Program

To provide a comprehensive mechanistic overview, we integrated the consistently differentially expressed genes into a coherent signaling framework, including key molecules in these molecular processes (Figure 5A). When embedding the consensus DEGs from T-early in their appropriate signaling network, six out of eight candidate genes are predominantly involved in coordinating the control of cell-cycle progression into S-phase and the assembly of the pre-replication complex, which is tightly controlled by active E2F (Figure 5A). The minichromosome maintenance (MCM) helicase protein complex consists of MCM2-7 and is part of the licensing of replication origins [25]. This is a tightly regulated multi-step mechanism, whereby the MCM2-7 helicase is loaded onto replication origins by the ORC1-6 complex, CDT1, and CDC6 [25,26,27,28]—all found in our analysis. Upon S-phase entry, re-replication is prevented by ubiquitination of CDT1 in a PCNA-dependent manner and through regulation of CDC6 stability [29,30]. Origin firing is subsequently triggered by Dbf4-dependent kinase (DDK)-mediated phosphorylation of the MCM complex, facilitating the recruitment of CDC45 and the GINS complex to form the active CMG helicase [29,31]. Interestingly, downregulation of the eight DEGs confirms observations that CDK4/6 inhibitors repress E2F function downstream of RB hypophosphorylation [15].

DEGs emerging from T-intermediate are primarily involved in the regulation of mitotic entry and progression (Figure 5B). These candidates were distributed across several discrete mitotic sub-processes, including disassembly of the Golgi apparatus [32], disintegration of the nuclear lamina [33,34], spindle assembly [35,36], and chromosome segregation with PLK1 and CDK1-Cyclin B in central regulatory roles [36,37].

In conclusion, the transcriptomic meta-analysis revealed a multi-step response to CDK4/6 inhibition, characterized by an early blockade of the G1/S-transition followed by an intermediate secondary step that inhibits an M-phase-related gene expression program.

### 2.6. Cross-Omic Comparison Identifies Conserved Candidates Mediating the Response and Resistance to PD

Next, we applied a cross-omic list comparison approach to determine whether the recurring DEGs from the transcriptomic analysis are also represented across independent experimental platforms like proteomics and functional genomics. Overlapping candidates could provide a list for further functional and clinical validation.

Overlaps of transcriptomic- and proteomic-derived lists would suggest functional biomarkers rather than transcriptional fluctuations within the range of biological variance. Integration of CRISPR gain-of-function (GoF) and knock-out (KO) data enabled the distinction between markers for sensitivity or resistance to treatment. For this analysis, we additionally integrated five proteomics and eight CRISPR screens (for inclusion criteria, see Section 4) for a cross-omic list comparison (Supplementary Table S8). Multi-set intersection testing was performed to evaluate if cross-omic list overlaps exceed chance expectation (Supplementary Table S9).

Four out of five proteomic datasets were derived from PD-sensitive cells that show growth inhibition in response to PD treatment [18,38,39,40]. We performed a meta-analysis, similar to the transcriptomics meta-analysis, to identify recurring differentially expressed proteins (DEPs). A core signature of 44 DEPs was present in at least two out of four studies (Supplementary Table S10) and will be referenced as P-response. Transcript–protein concordance was assessed using hypergeometric enrichment and Fisher’s exact test and confirmed significant enrichment beyond chance for concordantly differentially regulated genes and a strong association across protein and transcript levels (Supplementary Table S11).

The fifth proteomics dataset, with 199 DEPs originating from PD-resistant cells [41], was included to identify drivers of resistance and will be referenced as P-resistance. As P-resistance is derived from a single dataset, it cannot provide a conserved resistance signature, but yields a list of candidates likely involved in conferring resistance to CDK4/6 inhibition.

Further, we curated CRISPR KO screen data from seven publications, comprising eleven datasets with depleted sgRNAs and eight with enriched sgRNAs (Supplementary Table S8). Nine shared depleted sgRNAs were identified in at least seven out of eleven datasets [42,43,44,45,46] (Supplementary Table S12), which will be referred to as C-KO. Since loss of function for these candidates resulted in sgRNA depletion, they likely act as sensitizers to PD treatment.

Finally, to validate potential drivers of resistance to PD, we included enriched sgRNAs from a CRISPR GoF screen performed by our group [47], herein referred to as C-GoF.

These proteomic and functional genomic datasets were compared to T-consensus (Figure 3B) as well as the consensus signatures for T-early, T-intermediate, and T-late. Unlike in the transcriptomic meta-analysis, the proteomic and CRISPR meta-analyses did not account for the duration of treatment.

All integrated proteomic and CRISPR candidate lists exhibited overlaps with T-consensus (Figure 6A). Notably, RRM2 and members of the MCM2-7 complex were represented across multiple datasets, reinforcing the central role of inhibition of DNA replication in the cellular response to PD.

T-early consensus overlapped with the signatures of P-response, P-resistance, and C-KO (Figure 6B). While each comparison had only one single overlapping gene, the candidates were functionally centered around DNA replication (DTL, MCM2, and PCNA).

T-intermediate consensus showed overlaps with all other signatures except C-KO (Figure 6C). RRM2 was the primary common factor among all comparisons, alongside KIFC1 as a representative for secondary suppression of the mitotic gene expression program.

T-late consensus displayed the least connectivity, with PRIM1 as the sole overlapping candidate with the proteomic resistance dataset (Figure 6D). This divergence at the later time points suggests that the long-term response phenotype might be highly individual and cell line–specific.

Besides that, there are several genes—like RRM2 and members of the KIF and MCM families—that exhibited overlaps among the non-transcriptomic datasets (Figure 6E). This further highlights their central role in the cellular response to PD treatment regardless of temporally coordinated transcriptomic changes.

### 2.7. Cross-Omic Comparison Identifies a List of Conserved Genes That Mediate Response and Resistance to CDK4/6 Inhibition Across Different Tumor Types

Finally, we combined the results from the transcriptomic meta-analysis with the findings from the cross-omic comparison (Figure 7A). The first candidate list comprised the consensus DEGs within T-early, T-intermediate, and T-late—supplemented by functionally related genes described in the literature—that drive the initial G1-arrest and secondary M-phase blockade. This 20-gene selection was cross-referenced with the 28 overlapping genes between transcriptomic and proteomic comparisons, as well as the seven candidates overlapping between the transcriptomic meta-analysis and the CRISPR screens.

CDK2, DTL, and MCM2 were identified through the transcriptomic meta-analysis and further validated by proteomic data, while PCNA emerged as the sole factor that was validated with CRISPR screens. KIFC1, MCM6, and RRM2 showed consistent representation in transcriptomics, proteomics, and CRISPR screens.

After combining the results of the meta-transcriptomic analysis and cross-omics comparison, we identified a panel of 50 genes (Supplementary Table S13) for further experimental validation and clinical investigation as markers for response to palbociclib. The panel covers candidate genes that are functionally involved in the regulation of eight different aspects: cell-cycle regulators (CDK1, CDK2, CCNA1, CCNE1, CCNAB1, CDC25A, PBK, and PLK1); transcriptional regulation (DTL, E2F1, GLI1, MYC, RB1, RBL1, and ZNF367); licensing of DNA replication (CDT1, CDC6, ORC1, and MCM2-7); nucleotide supply and elongation during DNA replication (PCNA, PRIM1, RFC4, RRM2, TYMS, and WDHD1); DNA repair (MSH2 and RECQL4); chromatin restructuring (ASF1B, ATAD2, HELLS, NASP, and WDR76); nuclear structure and chromosome condensation (NCAPG, PRC1, TOP2A, TMPO, and SPTBN2); mitotic spindle organization and chromosome segregation (CDC10, CENPF, CENPQ, NUSAP1, KIFC1, KIF11, and KIF23).

## 3. Discussion

In this study, we hypothesized that an improved understanding of the central signaling pathways mediating therapy response to the CDK4/6 inhibitor palbociclib might allow for the identification of targets for promising combination therapies. The present study explicitly accounted for the influence of genetic heterogeneity on the therapeutic response by incorporating a broad range of cell lines and multi-omics data. This approach enables the identification of conserved molecular mechanisms underlying the response to PD treatment. Compared with previous studies that mainly focused on individual cell lines, single omics platforms, or isolated treatment durations, our integrative multi-omics approach provides a broader overview of the conserved transcriptional programs underlying CDK4/6 inhibitor response across different experimental settings. We included several studies using predefined inclusion criteria. We are aware that our design might not cover all data published regarding this question. Also, some of the curated publications only provide a single cell line, while no cell line is represented in two or more datasets, which complicates separation of real cell line–specific effects from methodological biases. Cell lines were deliberately selected for sensitivity to PD and could, therefore, not represent processes in cells showing innate resistance to CDK4/6 inhibition. Further, standardized quality metrics like read depth were not consistently reported and, therefore, could not be systematically assessed. While FDR and DEG counts were used as indicators of dataset quality instead, we cannot exclude the possibility that unmeasured differences in data quality contribute to variability across datasets. Another limitation of this study is the lack of available data on the phosphorylation level of RB and the effect of the PD concentration used in the publications included.

However, despite these limitations, the results display a panel of overlapping genes across different cell lines and experimental platforms and extend our knowledge of the molecular key events in the temporal kinetics of molecular changes during response to PD treatment. Since the analysis was restricted to data derived from palbociclib-treated cell lines, it remains to be determined whether the signature generalizes to other selective CDK4/6 inhibitors like ribociclib and abemaciclib.

The consensus signature for DEGs across all transcriptomic datasets confirmed a spatially separated multi-step process during the response to PD previously published by our group [16]. The present analysis builds upon this earlier model, using it as a framework to interpret an expanded collection of datasets. We acknowledge that the original T24 data used for creating the model were included again for this analysis, but they constitute only 3 out of the 27 sub-datasets analyzed here. However, we do not consider this to undermine our conclusions. As shown in Figure 2A, the majority of the independent datasets fit the proposed temporal model as well as or better than the original datasets from which the model was derived. This observation indicates that the model generalizes beyond the data used to establish it.

When combining a network analysis and pathway enrichment across temporal subgroups, we could refine the previously proposed three-step model [15] and describe molecules essential in mediating therapy response across a wide panel of different cell lines. The most prominent effect was the primary G1/G0-arrest, followed by a secondary differential expression of DEGs that regulate progression through the M-phase, supporting our previously proposed multi-step model of response to CDK4/6 inhibition [15]. Both, G1-arrest and inhibition of M-phase-related gene expression have been described before. However, we show these changes in a sequential context instead of only studying one of these two steps individually [2,48,49]. Crozier et al. (2023) also proposed a two-step model, describing two sequential waves of p21 activity: the first triggers G1-arrest in a p53- and MAPK-dependent manner, followed by G2 arrest induced by replicative stress and cell overgrowth [50].

Since bulk RNA-sequencing cannot distinguish between direct inhibition of M-phase progression and a depletion of the mitotic fraction, slowed down proliferation, or DREAM/E2F-mediated transcriptional repression, further experimental investigation will be necessary to unravel the secondary response to CDK4/6 inhibition.

Based on our findings and the literature context, we propose a refined version of the previously proposed multi-step model of response to PD treatment: The initial phase of therapy response is characterized by immediate inhibition of G1/S-transition, including the assembly and activation of the pre-replication complex and the subsequent inhibition of DNA replication (Figure 5A and Figure 7B). With prolonged exposure to CDK4/6 inhibitors (24 h), a secondary response is triggered, which is characterized by the suppression of gene expression involved in mitotic entry and progress. This response includes the disruption of nuclear envelope breakdown, Golgi trafficking, and cytokinesis, which likely reinforces cell-cycle arrest. Combination approaches that specifically support this phase of the therapeutic response have been described, for example, through the inhibition of AURKA or TTK [51,52]. The prolonged therapy response (>48 h) is characterized by sustaining the previously observed transcriptional signatures, specifically for G1-arrest, suppression of the DNA replication machinery, and the blockade of M-phase progression.

The continuity of these signaling processes suggests that the cellular status is still mainly characterized by the inhibition of cell-cycle progression. Therefore, the synergistic combination approaches designed to support the early and intermediate phases of PD response remain mechanistically relevant.

Treatment of patients with CDK4/6 inhibitors is, in general, more potent when applied in combination with additional drugs. There are several combination therapies described that support the PD-mediated G1-arrest and prevent reentry in the cell cycle. CDK4/6 inhibition can be circumvented at the cellular level by PI3K-signaling-mediated overexpression of Cyclin D and subsequent reactivation of CDK4/6 [48,53]. Therefore, upstream regulatory receptor tyrosine kinases, as well as central proteins of the PI3K and MAPK signaling pathways, are reasonable targets for combination therapy with CDK4/6 inhibitors. Also, the complementary inhibition of CDK2 has been suggested as a suitable strategy [54]. We identified the Ephrin-receptor family as a novel element contributing to the response to CDK4/6 inhibitors. However, this remains a cell line–specific observation, since Ephrin-receptors were not identified as differentially regulated upon CDK4/6 inhibitors in any other dataset.

Integrating transcriptomics with complementary approaches such as proteomics or functional genomics is an established strategy for characterizing treatment response [55]. Previous cross-omics comparisons of PD treatment have consistently identified cell-cycle arrest to be the primary effect [18,49], yet most studies focus on single cell lines or one time point only. Our cross-comparison of consensus sequences—derived from transcriptomics, proteomics, and functional genomics—addresses this gap and integrates data across different cell lines and treatment durations to identify the core conserved mechanisms of response.

The high number of response genes (overlap in transcriptomics–proteomics data) and the comparably low number of resistance genes (overlap in transcriptomics–CRISPR screens) could be attributed to a key weakness of bulk RNA sequencing: less prominent but biologically meaningful results—like highly diverse resistance mechanisms—can be hidden behind more dominant transcriptional effects. CRISPR screens themselves yield highly variable top candidates for resistance markers depending on the cancer type, cell line, or screen design [42,43,44,45,46,47], which complicates determining a consensus gene set. Resistance mechanisms to PD treatment are particularly heterogeneous. They are cell line–specific, develop over time, and vary not only across patients and cell lines but also temporally within a single resistance population [56,57,58]. Distinct genetic and epigenetic backgrounds shape escape mechanisms under sustained CDK4/6 inhibition, even when they share the primary response of cell-cycle arrest [58]. Single-cell analysis across PD-sensitive and -resistant breast cancer cell lines confirmed high intra- and inter-cell line heterogeneity in the expression of canonical resistance markers [58], which further complicates the identification of common mechanisms and cross-platform validation.

This heterogeneity likely also explains our low number of consensus genes identified for T-late and the lack of typical resistance genes. Also, the response mechanisms appear to be diverse. Prolonged PD treatment is reported to drive cells into senescence-like or quiescent states [15], and the resulting transcriptome signatures are highly specific to the inducing stress factors. Moreover, the senescent phenotype is dynamic and evolves at varying intervals after induction [59,60], so only the most conserved transcriptional changes are expected to persist across datasets.

Despite lacking a direct functional relationship, the three identified genes in the T-late consensus each reflect the cellular processes that maintain the response to PD treatment. Downregulation of DNA primase subunit 1 (PRIM1) and upregulation of the transcriptional repressor MXD4 are consistent with the observed G1-arrest following treatment. The downregulation of nuclear lamina filament LMNB1 reflects the disintegration of the nuclear lamina during M-Phase progression and is typically associated with senescence [60].

Interestingly, our analysis did not identify previously described mediators of resistance to CDK4/6 inhibition, such as FGFR, EGFR, PI3K, AKT, or mTOR [56,61,62]. However, the candidates E2F1, PLK1, and RRM2 have previously been described in this context [40,61,62]. This indicates that resistance genes are probably representing a heterogeneous group, defined by a specific genetic background. The genes identified in our analysis represent conserved molecular changes associated with the response to therapy. Thus, stratification of patients based on aberrant molecular changes in upstream elements that regulate activation of the identified processes might be useful for deciding on an individualized combination therapy approach. However, a thorough experimental and, ultimately, clinical validation of the gene signature is required to assess whether it can be transferred into clinical application.

In summary, our study provides a comprehensive cross-omics comparison of the conserved molecular response to CDK4/6 inhibition across multiple cancer models. By integrating transcriptomic, proteomic, and functional genomic datasets, we further refined the previously proposed multi-step response model and identified a panel of highly conserved gene signatures. However, the clinical value of these findings must be validated by applying predictive modeling, receiver operating characteristic analysis, or assessment of signature performance in classifying responders versus nonresponders.

## 4. Materials and Methods

### 4.1. Cross-Omics Comparison of Transcriptomics, Proteomics, and CRISPR Screen Data

All transcriptomic [15,18,19,20,21,22,23,24], proteomic [18,38,39,40,41], and CRISPR [42,43,44,45,46,47,63,64] screen data employed were published previously.

Datasets for the transcriptomic meta-analysis were sourced from Kositza et al. (2023) [65]. Inclusion criteria were monotherapy with PD for 6–72 h and available, preprocessed differential expression data (log2FC, FDR). If several PD concentrations were available, the highest was included. DEGs were filtered for an |log2FC| > 1 compared to untreated 0 h control and a false discovery rate (FDR) of <0.05. The direction of differential expression was assessed for each gene across each dataset. Only genes with consistent direction were included in the analysis. Datasets were grouped by duration of treatment (6–8 h, 24 h, and 48–72 h) and analyzed across and within these groups for overlapping DEGs (Supplementary Table S8). Multi-set intersection testing was applied using the SuperExactTest package (version 1.2.0) to test whether the observed overlap exceeded chance expectation [66] (Supplementary Table S14).

Independent proteomics and CRISPR screen datasets were curated with a PubMed search using the terms “Proteomics AND Palbociclib” (13 March 2026) or “CRISPR screen AND Palbociclib” (26 March 2026). General inclusion criteria were that the data be publicly available, preprocessed, and derived from cell culture and monotherapy with PD. sgRNAs were divided into upregulated and downregulated and processed separately. Cutoffs for each dataset are summarized in Supplementary Table S8. sgRNA overlaps were analyzed using upset plots, and depleted sgRNAs that were represented in seven out of eleven datasets were defined as consensus C-KO. Since only one GoF CRISPR screen was available, all enriched sgRNAs were included in the cross-omics analysis.

For the CRISPR screen data, we included knock-out screens as well as one gain-of-function screen from PD-sensitive cell lines (Supplementary Table S8).

For proteomics, we included results from both PD-sensitive cells—that show growth inhibition in response to PD treatment—and resistant cell lines. Inclusion criteria were that the data be publicly available, preprocessed, and derived from cell culture and monotherapy with PD. Cutoffs for each dataset are summarized in Supplementary Table S7. DEP overlaps were analyzed using upset plots, and DEPs represented in two out of four datasets were defined as P-response.

The resulting consensus gene signatures were compared with each other and with the lists resulting from the transcriptomic meta-analysis. Overlaps in candidate lists were analyzed using upset plots. Directional concordance between DEGs and DEPs was assessed using hypergeometric enrichment and Fisher’s exact test. To formally test whether the observed cross-omics list overlaps exceed what can be expected by chance, we performed multi-set intersection testing using the SuperExactTest package [66] (Supplementary Table S11). Candidates that were present in transcriptomics and at least one other omics type were collected in one list and compared to the transcriptomics-only list. Supplementary Figure S4 provides a graphical overview of this analysis strategy.

### 4.2. PERMANOVA Analysis of Transcriptomic Datasets

PERMANOVA was performed using the vegan package (version 2.7-6) in R [67]. We used a nested model with 999 permutations to fit our data structure (Publication/Cell line). We also conducted a dispersion test, but since several datasets are represented by only one cell line/publication, these groups show a distance to centroid of 0.0. We believe this test produces a mathematical artifact rather than evidence of meaningful biological within-group variability.

### 4.3. STRING Protein–Protein Interaction Network

Protein–protein interaction networks were created using the STRING database (protein.links.full.v12.0) [68], displaying only physical interactions with an interaction score > 400. Networks were modified, mapped with expression data, and clustered using Cytoscape (version 3.10.3) [69]. If possible, singletons were connected to the largest subnetwork with the stringApp expand network function (versoin 2.2.0).

### 4.4. Pathway Enrichment Analysis

Pathway enrichment analysis was conducted in R (version 4.3.3) using clusterProfiler (version 4.10.1) and ReactomePA (version 1.46.0) [70,71]. Gene lists derived from the STRING PPI network analysis (6–8 h and 24 h) or T-consensus were converted to Entrez IDs (org.Hs.eg.db version 3.18.0) with a mapping success rate of 96.4% and used as the input for the pathway enrichment analysis. Human gene sets defined by the Reactome database (version 94 for T-early and T-intermediate, and version 96 for T-consensus) were used as a reference for the enrichment analysis [72]. The background gene universe was org.Hs.eg.db [73]. Statistical significance was assessed with Benjamini–Hochberg. Redundant terms were not collapsed and are reported as returned by ReactomePA.

### 4.5. Cell Culture and Inhibitors

T24 bladder cancer cells from ATCC (Manassas, VA, USA) were cultivated in DMEM high-glucose medium containing 10% FBS and 1% Penicillin/Streptavidin at 37 °C and 10% CO_2_.

Palbociclib isethionate (PD-0332991, PD, MedChemExpress LLC (MCE), Princeton, NJ, USA) was dissolved in sterile water for a 10 mM stock solution and stored at −20 °C. ALWII-49-7 was obtained from MCE (Princeton, NJ, USA) and dissolved in DMSO for a stock concentration of 1 mM and stored at −80 °C. Working concentrations were freshly prepared. Controls reflect the highest DMSO concentration used in the assay.

### 4.6. Quantitative Real-Time Polymerase Chain Reaction

T24 cells were treated with the indicated concentrations of inhibitors and harvested after 4, 8, or 24 h. Total RNA was extracted using the mirVana miRNA Isolation Kit (Thermo Fisher Scientific, Waltham, MA, USA). cDNA was synthesized from 2 µg of RNA using a high-capacity cDNA reverse transcription kit (Thermo Fisher Scientific, Waltham, MA, USA). qPCR was conducted with SYBR Green Master mix (Thermo Fisher Scientific, Waltham, MA, USA) and the CFX96 Real-time PCR detection system (Bio-Rad Laboratories, Inc., Hercules, CA, USA). The ΔΔCT-Method was used for relative quantification, with Actin as the endogenous reference gene. The primers used for qPCR are listed in Supplementary Table S15.

### 4.7. ZIP Model

The nature of interaction of two compounds was assessed using the Zero Interaction Potency Model (ZIP) [74] and the online tool “Synergy finder” (version 3.0).

### 4.8. Software

Data pre-processing, analysis, and visualization were conducted using R (version 4.3.3) and RStudio (version 9.2.191.153). Heatmaps and upset plots were created using the ComplexHeatmap package (version 2.18.0) [75].

## Figures and Tables

**Figure 1:**
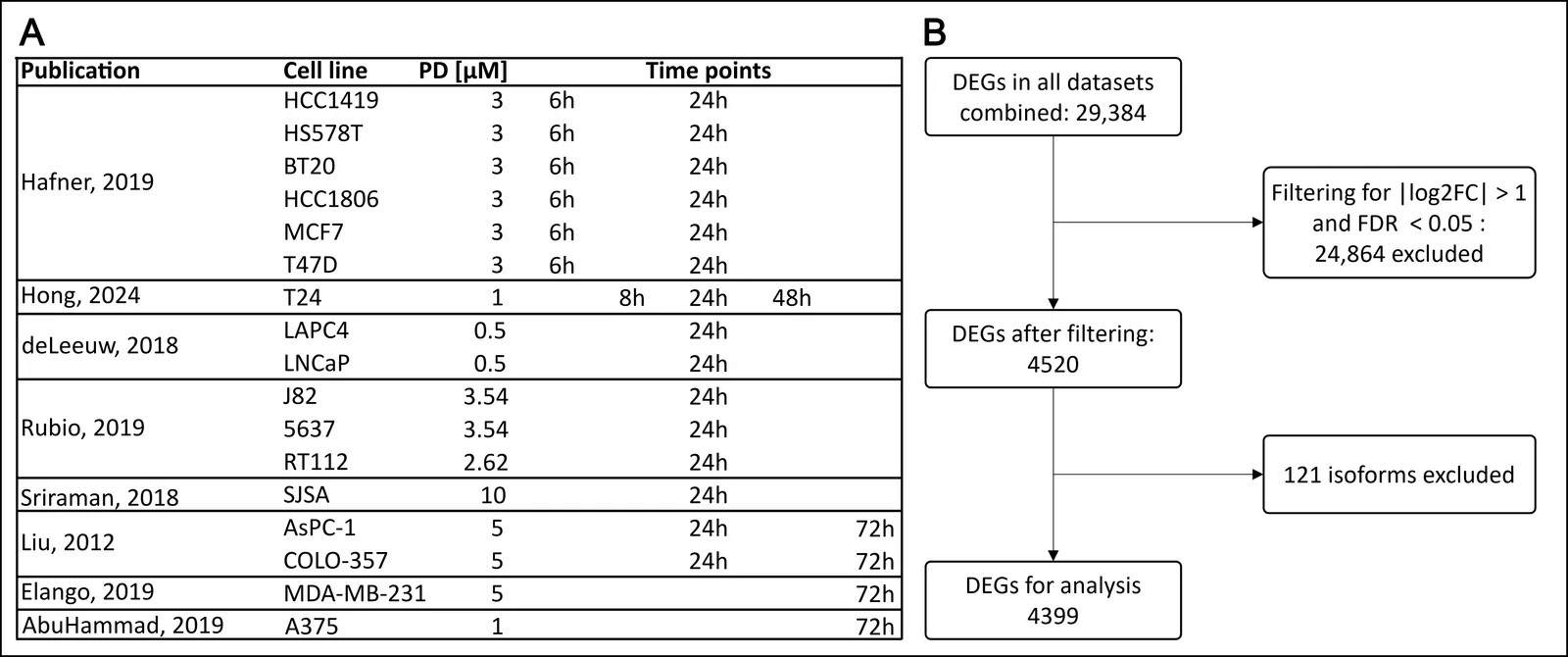
(**A**) Overview of PD response transcriptomic datasets used for meta-analysis [15,18,19,20,21,22,23,24]. (**B**) Filtering strategy for transcriptomic data prior to meta-analysis.

**Figure 2:**
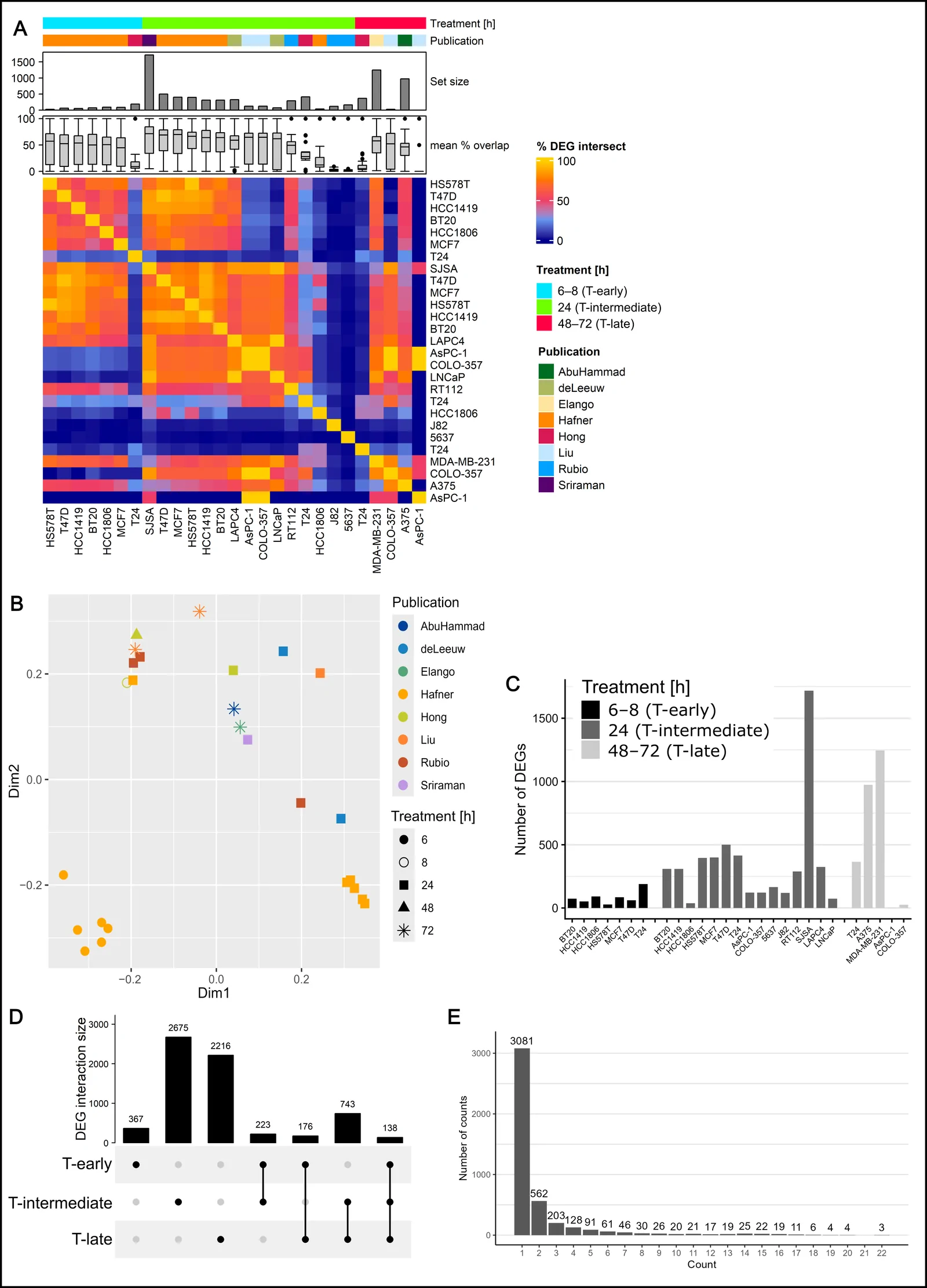
Transcriptional response to PD is organized into three consecutive phases: (**A**) Proportional overlap of DEGs, calculated relative to the smaller dataset of each comparison, with the boxplot top annotation showing mean % overlap of each dataset with all other datasets, and the bar plot top annotation showing number of DEGs per dataset. Datasets were organized primarily by duration of PD treatment and secondarily by descending mean percent overlap in DEGs. (**B**) Principal coordinates analysis of transcriptomics datasets. (**C**) Number of DEGs in each dataset after filtering. (**D**) Combined number of DEGs in time point subgroups and number of overlapping genes. (**E**) Number of sets a DEG is represented in was assessed, displaying the distribution of counts.

**Figure 3:**
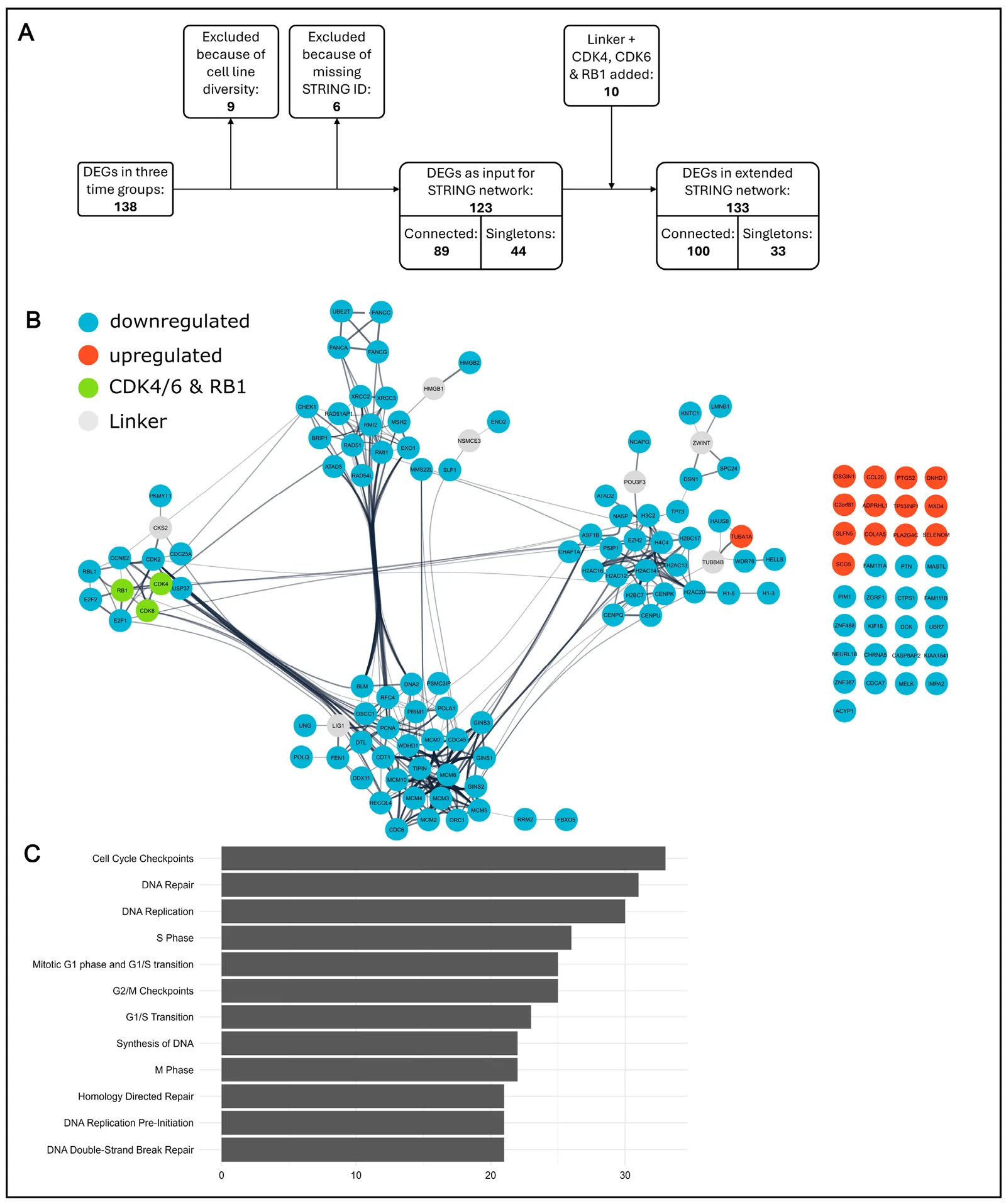
Consensus genes across multiple tumor cell lines and treatment durations are functionally connected and focus on cell-cycle regulation, DNA replication, and repair: (**A**) protein–protein interaction network strategy; (**B**) STRING protein–protein interaction network based on T-consensus; (**C**) pathway enrichment analysis of T-consensus.

**Figure 4:**
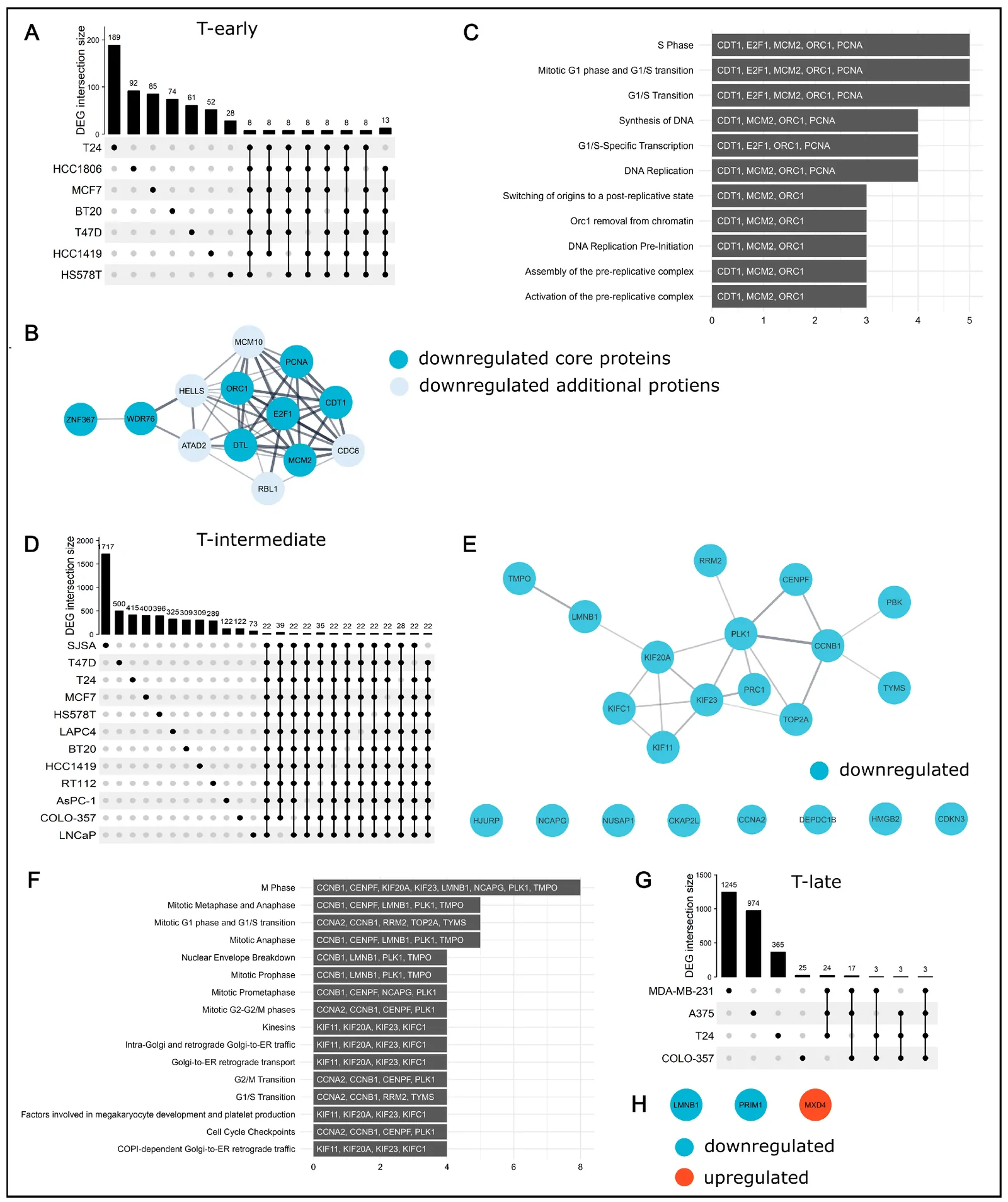
Functional focus of early and intermediate response shifts from inhibition of S-phase transition and DNA replication to inhibition of M-phase: (**A**) upset plot showing intersecting DEGs between the datasets in T-early; (**B**) STRING protein–protein interaction network of common DEGs in T-early; (**C**) pathway enrichment analysis of common DEGs in T-early; (**D**) upset plot showing intersecting DEGs between the datasets in T-intermediate; (**E**) STRING protein–protein interaction network of common DEGs in T-intermediate; (**F**) pathway enrichment analysis of common DEGs in T-intermediate based on Reactome terms; (**G**) upset plot showing intersecting DEGs between the datasets in T-late; (**H**) STRING protein–protein interaction network of common DEGs in T-late.

**Figure 5:**
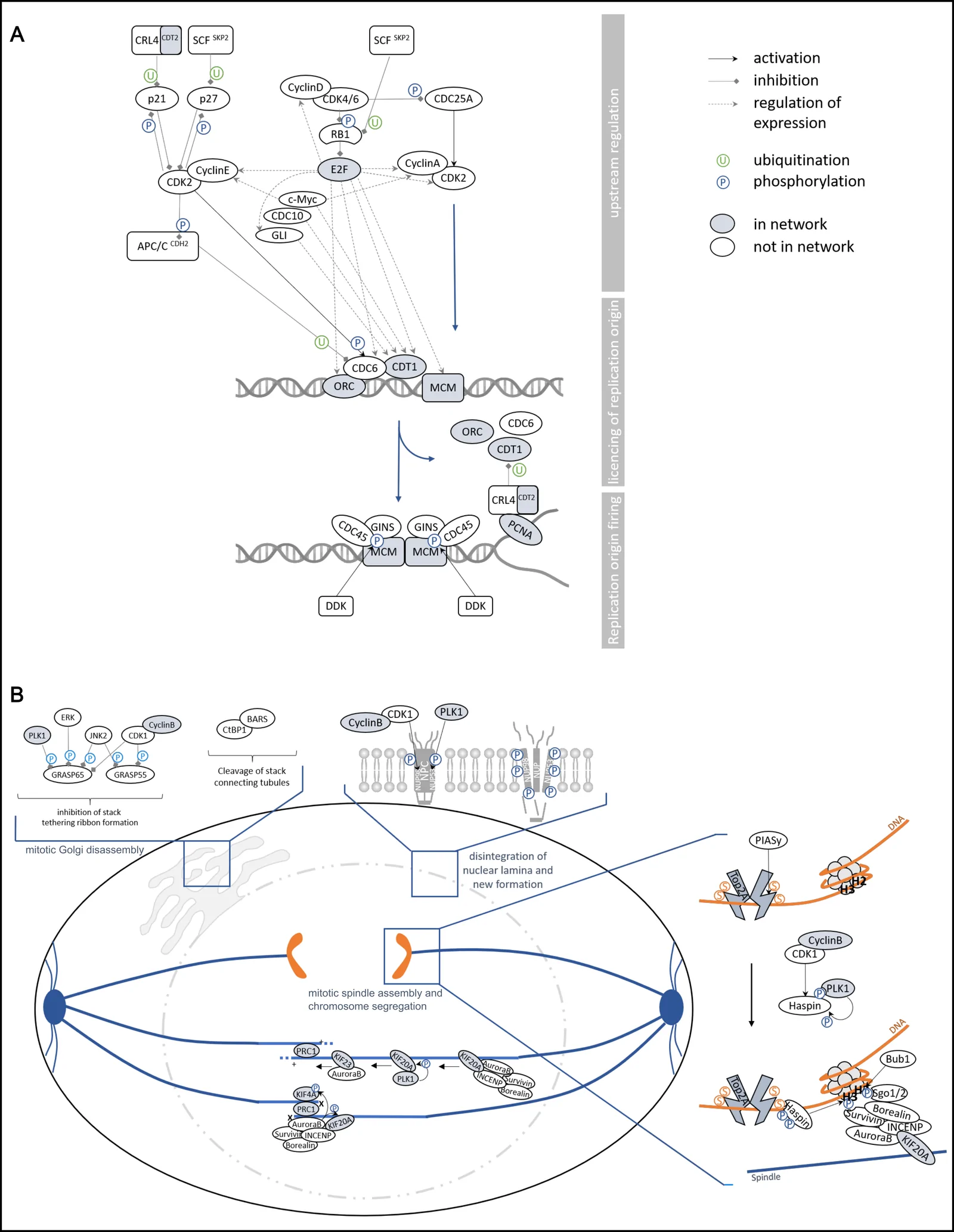
Graphical overview of molecular functions of T-early consensus and T-intermediate consensus: (**A**) Functional connection of T-early consensus and upstream regulators relevant for induction of therapy response: regulation of S-phase entry and origin of replication licensing and firing. (**B**) Functional connection of T-intermediate consensus. Blue proteins: DEGs are part of the transcriptomics meta-analysis and STRING protein–protein interaction network. White proteins: added based on the literature research.

**Figure 6:**
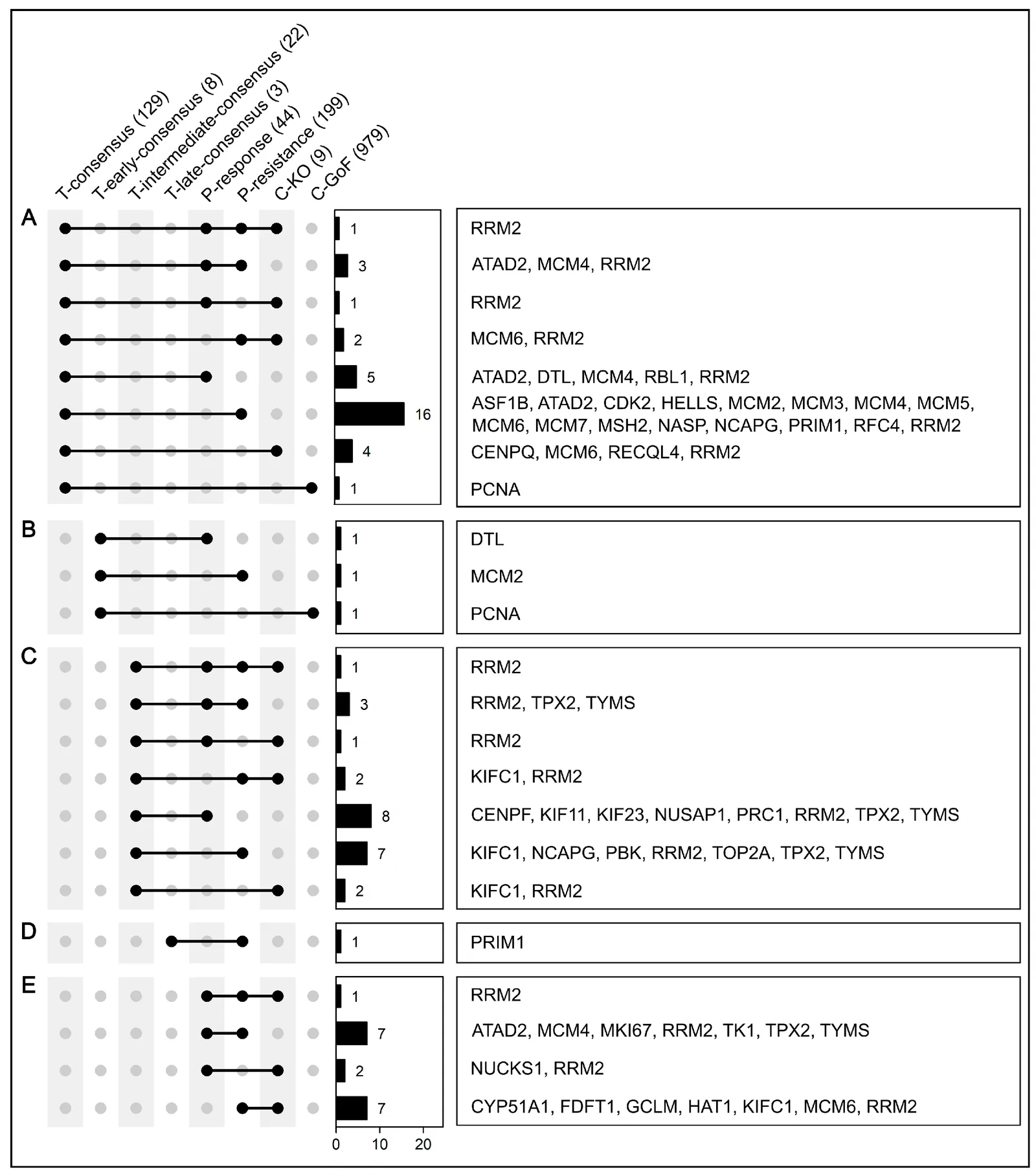
Cross-omic gene list comparison: comparison of transcriptomic, proteomic, and CRISPR screen data to identify robust candidates mediating response and resistance to PD: (**A**) overlaps between T-consensus and proteomic and CRISPR screen data; (**B**) overlaps between T-early and proteomic and CRISPR screen data; (**C**) overlaps between T-intermediate and proteomic and CRISPR screen data; (**D**) overlaps between T-late and proteomic and CRISPR screen data; (**E**) overlaps between proteomic and CRISPR screen data. Numbers behind signature names indicate number of DEGs/DEPs/sgRNAs.

**Figure 7:**
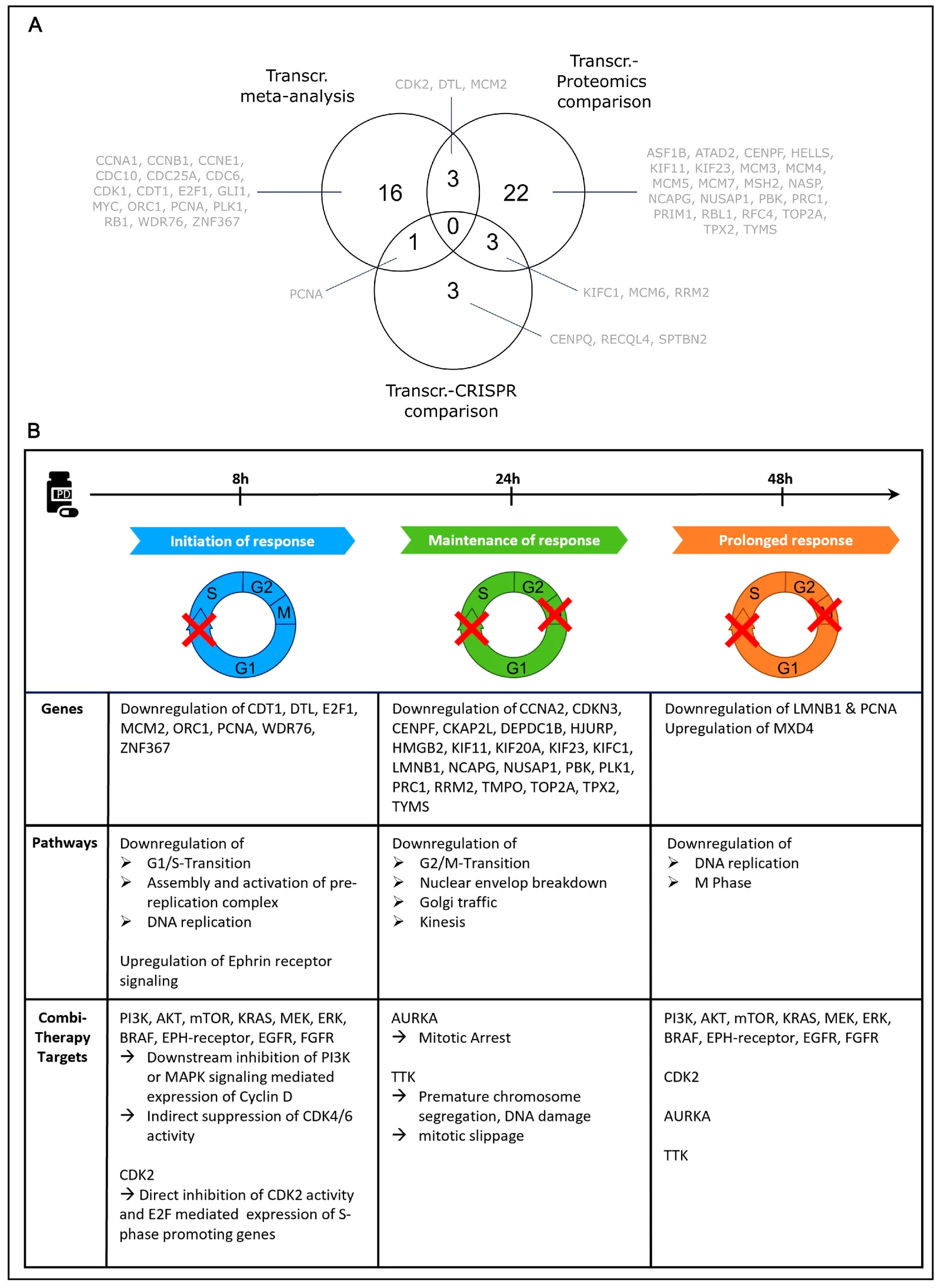
Comparison of different approaches to identify conserved mediators of response and resistance to PD treatment and refinement of multi-step model of PD response: (**A**) comparison of candidate lists based on transcriptomics meta-analysis (**left**) and cross-omics comparison between transcriptomic and proteomic signatures (**right**) and between transcriptomic and CRISPR screen signatures; (**B**) refined model of multi-step response to PD.

## Data Availability

The original contributions presented in this study are included in the article/Supplementary Materials. Further inquiries can be directed to the corresponding author.

## Supplementary Materials

The following supporting information can be downloaded at https://www.mdpi.com/article/10.3390/ijms27198507/s1.

## Abbreviations

The following abbreviations are used in this manuscript:

DEG	Differentially expressed gene
DEP	Differentially expressed protein
ET	Endocrine therapy
GoF	Gain of function
Log2FC	Logarithm to the base of 2-fold change
PCoA	Principal coordinates analysis
PD	Palbociclib
PPI	Protein–protein interaction
ZIP	Zero interaction potency model
