# Supplementary material for: Identification of a Conserved Temporal Molecular Response Signature to CDK4/6 Inhibition Through Multi-Omics Integration Across Cancer Cell Lines
Source: Int J Mol Sci. 2026 Sep 24;27(19):8507. doi: 10.3390/ijms27198507 (PMC13665524; doi:10.3390/ijms27198507)
Supplement: Supplementary file 1 [file ijms-27-08507-s001.zip › Supplementary_figures_revised.pdf]

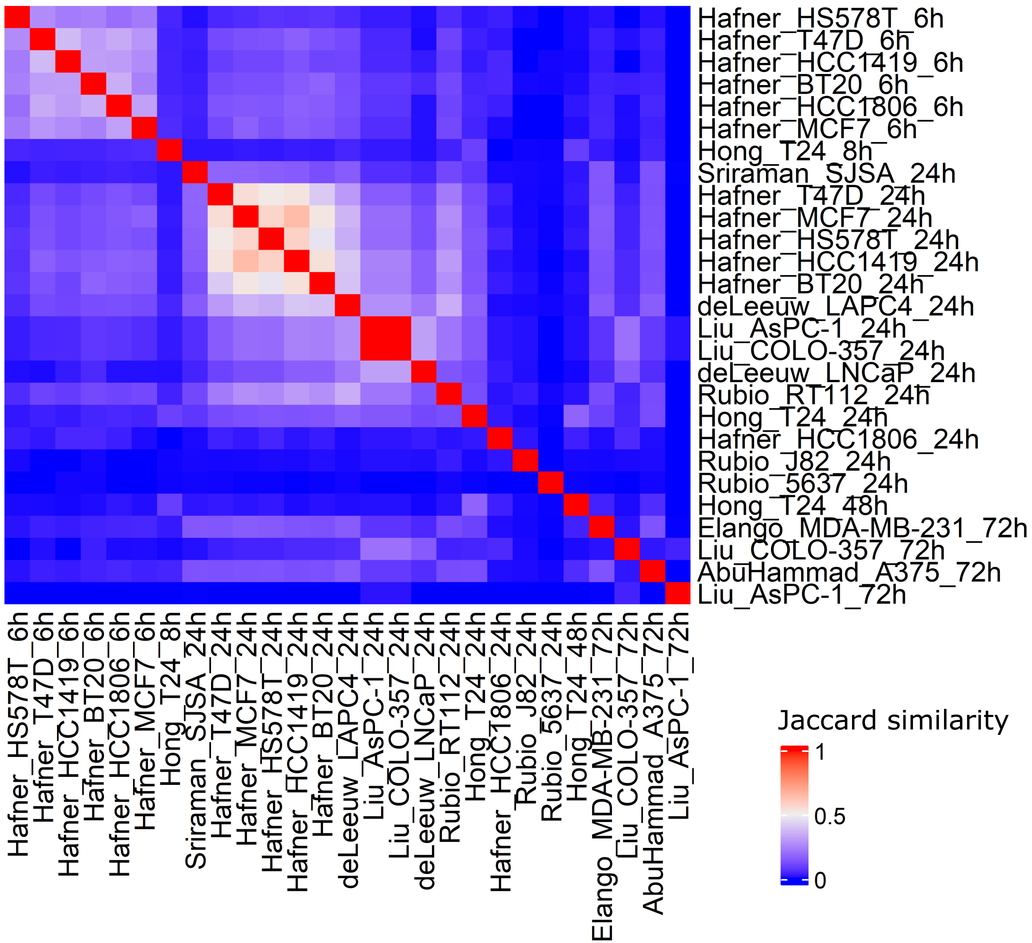

Supplementary Figure S1 Jaccard Similarity matrix of transcriptomic datasets

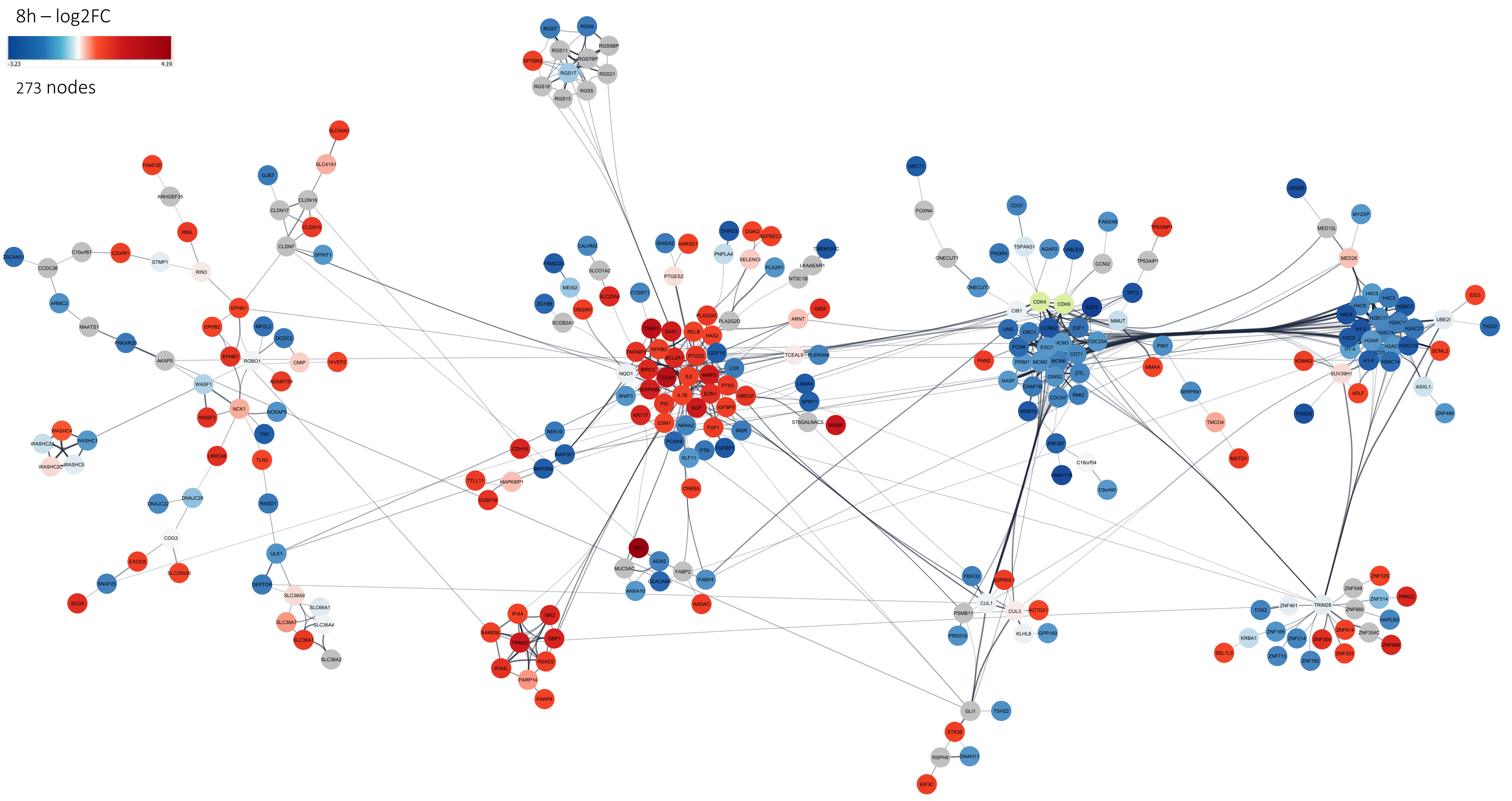

Supplementary Figure S2 T24 cells were treated with 0.5 µM PD and RNA was isolated 8, 16,20,24 and 48 hours after Treatment followed by RNA-sequencing (n = 3) (single-end Illumina). Differential gene expression analysis was performed to compare gene expression profiles after PD treatment to the 0h untreated control. The differential gene expression analysis of 8h of PD treatment compared to the untreated 0-hour control was filtered for genes with |log2FC| > 1 and p < 0.05. Subsequently, the STRING protein-protein interaction table was filtered for interactions were both interaction partners are part of that gene list and display an interaction score nodes was visualized with Cytoscape. Single nodes and smaller subnetworks were connected with the largest subnetwork by including linking proteins using the Cytoscape STRING extension and the expand network function. Nodes were grouped by protein function. Node color was mapped to the 8h log2 fold change. Proteins were clustered by function. Edges were bundled to increas clarity of the resulting network.

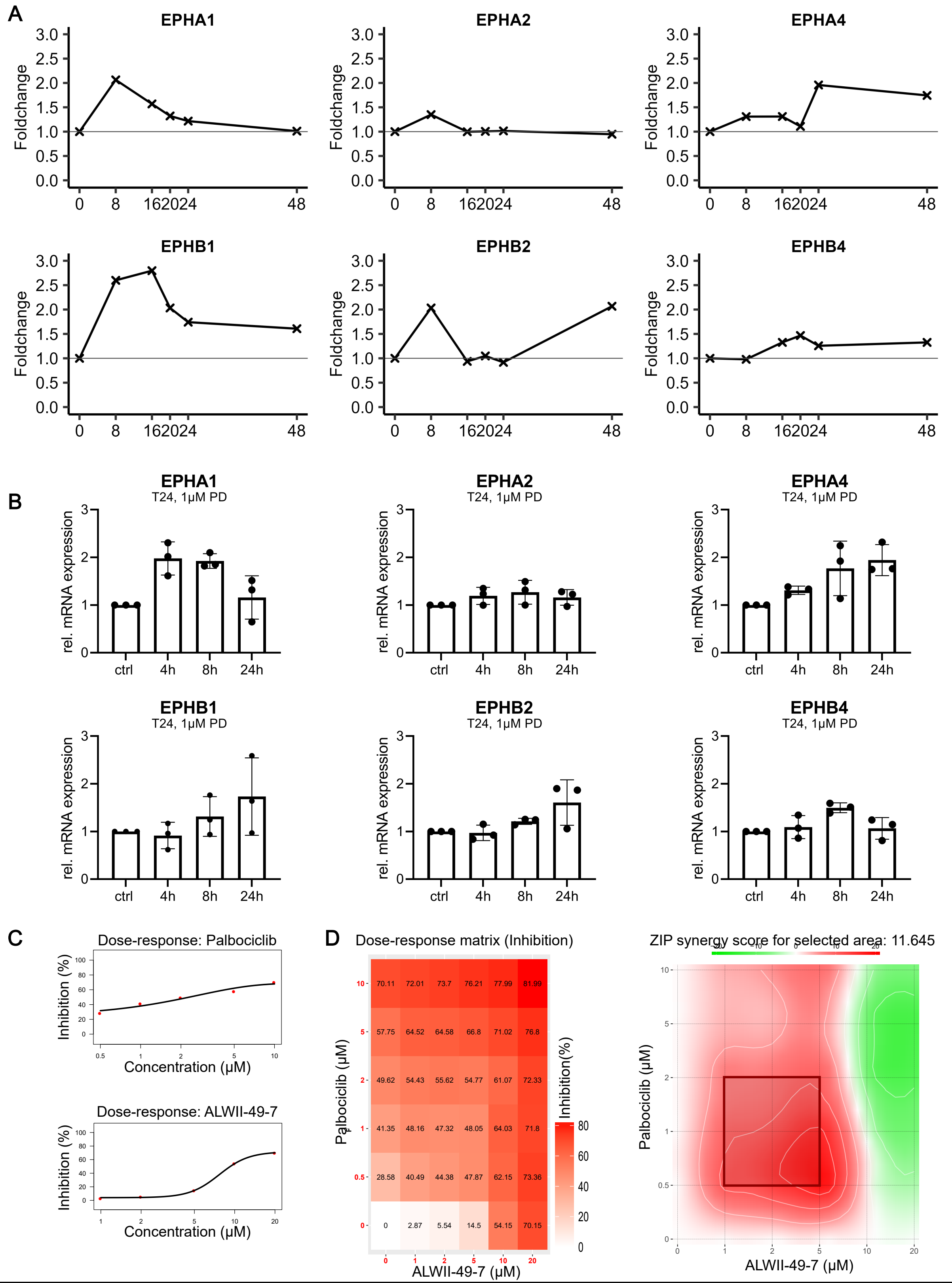

Supplementary Figure S3 (A) mRNA levels of ephrin receptors in T24 cells treated with 1µM PD for the indicated time compared to the untreated 0h control (single-end illumina mRNA sequencing), n=3. (B) T24 cells were treated with 1µM PD for the indicated time. mRNA expression of ephrin receptors was assessed relative to actin with qPCR, n=3, technical replicates = 3, error bars indicate standard deviation of the mean. (C) Dose response assessment of palbociclib and ALWII-49-7 monotherapy in T24 over 72 hours (n = 2, representative replicate displayed). (D) Dose response matrix of Palbociclib and ALWII-49-7. T24 cells were treated with the indicated concentrations of inhibitors for 72 hours (n = 2, representative replicate displayed) and a dose-response matrix was created and a interaction landscape was calculated using the ZIP synergy model. The ZIP score for the selected area (red square) is indicated.

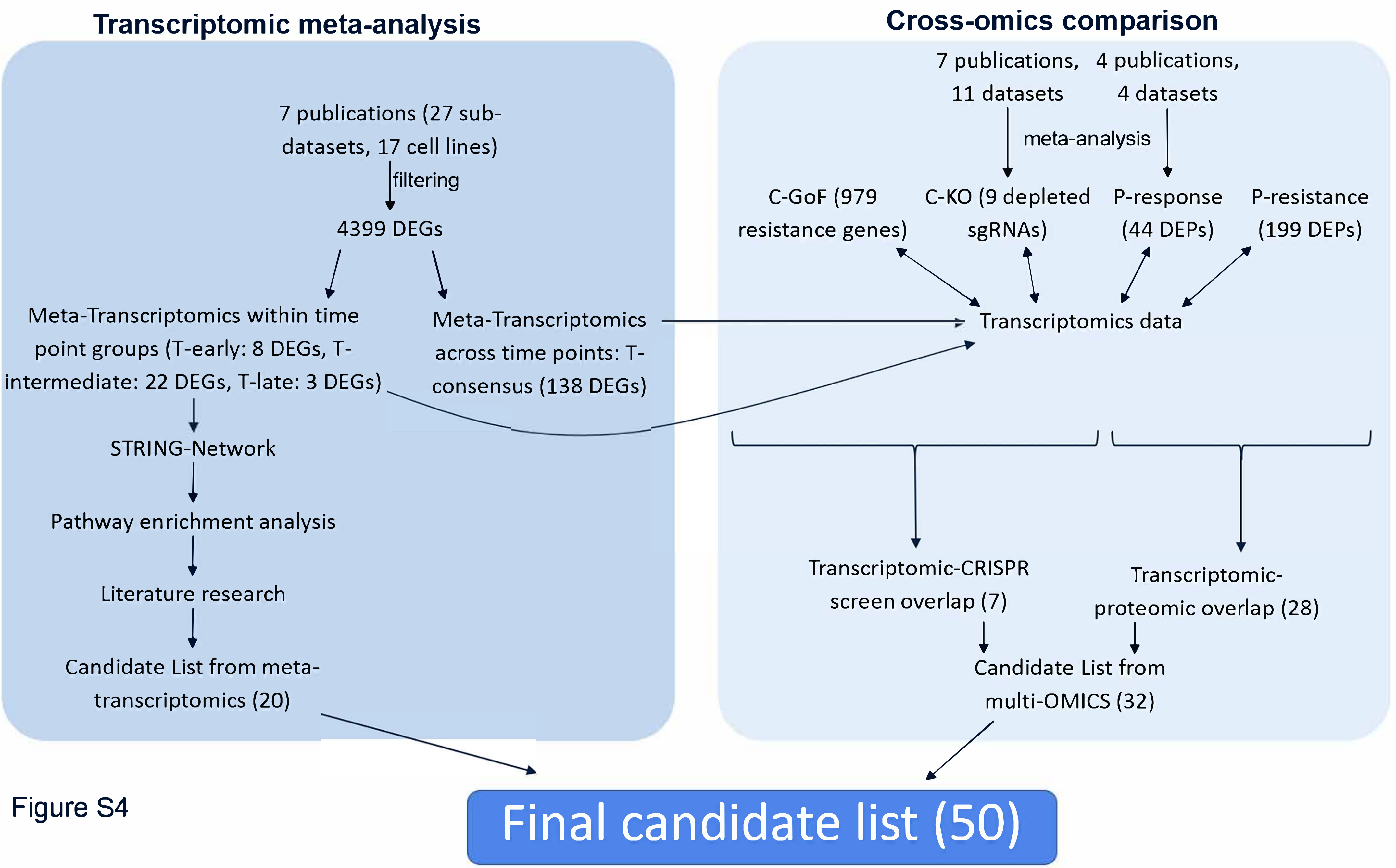
Transcriptomic meta-analysis
Cross-omics comparison
7 publications (27 sub-datasets, 17 cell lines)
filtering
4399 DEGs
Meta-Transcriptomics within time point groups (T-early: 8 DEGs, T-intermediate: 22 DEGs, T-late: 3 DEGs)
Meta-Transcriptomics across time points: T-consensus (138 DEGs)
STRING-Network
Pathway enrichment analysis
Literature research
Candidate List from meta-transcriptomics (20)
7 publications, 11 datasets
4 publications, 4 datasets
meta-analysis
C-GoF (979 resistance genes)
C-KO (9 depleted sgRNAs)
P-response (44 DEPs)
P-resistance (199 DEPs)
Transcriptomics data
Transcriptomic-CRISPR screen overlap (7)
Transcriptomic-proteomic overlap (28)
Candidate List from multi-OMICS (32)
Final candidate list (50)

Figure S4
